# Target Detection over the Diurnal Cycle Using a Multispectral Infrared Sensor

**DOI:** 10.3390/s17010056

**Published:** 2016-12-29

**Authors:** Huijie Zhao, Zheng Ji, Na Li, Jianrong Gu, Yansong Li

**Affiliations:** School of Instrumentation Science & Opto-Electronics Engineering, Beihang University, 37 Xueyuan Road, Haidian District, Beijing 100191, China; hjzhao@buaa.edu.cn (H.Z.); jizhengss1988@buaa.edu.cn (Z.J.); karon@buaa.edu.cn (J.G.); lysbuaa@buaa.edu.cn (Y.L.)

**Keywords:** infrared sensor, multispectral, diurnal cycle, thermal crossover

## Abstract

When detecting a target over the diurnal cycle, a conventional infrared thermal sensor might lose the target due to the thermal crossover, which could happen at any time throughout the day when the infrared image contrast between target and background in a scene is indistinguishable due to the temperature variation. In this paper, the benefits of using a multispectral-based infrared sensor over the diurnal cycle have been shown. Firstly, a brief theoretical analysis on how the thermal crossover influences a conventional thermal sensor, within the conditions where the thermal crossover would happen and why the mid-infrared (3~5 μm) multispectral technology is effective, is presented. Furthermore, the effectiveness of this technology is also described and we describe how the prototype design and multispectral technology is employed to help solve the thermal crossover detection problem. Thirdly, several targets are set up outside and imaged in the field experiment over a 24-h period. The experimental results show that the multispectral infrared imaging system can enhance the contrast of the detected images and effectively solve the failure of the conventional infrared sensor during the diurnal cycle, which is of great significance for infrared surveillance applications.

## 1. Introduction

Infrared imaging detection systems are becoming more prevalent in numerous fields, including remote sensing [1], medical monitoring [2], military surveillance [3], and scientific research [4,5]. These systems offer major advantages over visual detection systems, such as their continuous day and night imaging capabilities, especially for target detection and acquisition [6].

When targets are aimed to be detected over the diurnal cycle using a conventional mid-infrared (3~5 μm) sensor, the results are generally affected by thermal crossover, where the infrared image contrast from the target and the background is difficult to discriminate from each other as the target would have integrated with the background and the radiation difference between the target and background was too low to be sensed by the infrared thermal sensor. Moreover, this could cause the targets to be blended into the background, lowering the detection accuracy, and even make the thermal sensor lose the target. In addition, the thermal crossover may also occur at any point in the day, because of solar loading, clouds, rain and fog. Therefore, it is critical to solve this problem for the conventional mid-infrared thermal sensor, especially for the infrared surveillance system.

In the last few decades, research has focused on how to solve the problem of infrared detection during thermal crossover periods and the thermal polarization technique, which is proposed as a method to enhance conventional thermal imaging, has been employed. Felton et al. [7,8,9] compared the crossover periods for mid-and long-wave infrared polarimetric and conventional thermal imagery. The mid-infrared (3~5 μm) imaging polarimeter they used was based on a division-of-aperture (DoA) lens technology developed by Polaris Sensor Technologies, which employed a 2 × 2 array of mini-lenses followed by four linear polarizers at different orientations, forming four identical images of the scene on four quadrants of the sensor focal plane array. The long-wave infrared (8~12 μm) polarimeter they used was a microbolometer-based rotating retarder imaging polarimeter developed by Polaris Sensor Technologies, which could capture up to 12 images sequentially in time with each image at a different orientation. Their experimental results showed that the polarimetric technology could be used as a method to enhance the conventional infrared image contrast between the targets and background during thermal crossover periods. However, their infrared image contrast improvement was not direct but resulted from the calculation of Stokes vector formula, which might not be suitable for the systems that requires high-performance of real-time processing. Still, as their work mainly focused on polarimetric detection experiments, what the pictures are when the target integrated with the background during the diurnal cycle and the theoretical analysis on how the thermal crossover influences the conventional thermal sensor and why the polarimetric technology could be used to solve the thermal crossover detection problem was also not mentioned. Based on Felton’s research, Wilson et al. [10,11] used a single pixel scanning passive millimeter-wave polarimetric sensor, operating at a frequency of 77 GHz with a noise equivalent temperature difference (NETD) of 0.5 K, to measure the infrared image contrast during thermal crossover periods. As the passive millimeter-wave sensor is designed with capabilities to measure two linear polarization states simultaneously, it breaks the limitation that many of millimeter wave (mmW) sensors are only able to detect a single linear polarization state and improve the detection accuracy. Additionally, Retief et al. [12] studied the prediction method of thermal crossover based on imaging measurements under different weather conditions over the diurnal cycle. They used a series of infrared background objects images as the basis to establish the heat balance model and, on this basis, to predict when the thermal crossover may occur. In addition to the thermal polarization technique, the infrared multispectral technology is also considered as an important approach to solve the thermal crossover detection problem. The prior studies [13,14,15,16,17] on infrared multispectral technology mainly showed the potential benefits of infrared multispectral processing for clutter-limited ground target detection. However, due to constraints on the spectral resolution, band coverage, and radiometric sensitivity of existing sensors at that time, accurate measurement data and the real experimental image data were not available. Despite this, these studies firstly made the infrared multispectral technology a potential method for target/background identification. Furthermore, Schwartz and Eismann et al. [18,19,20] conducted a series of multispectral field measurements at Redstone Arsenal using a Bomem-developed high-sensitivity infrared Fourier Transform Spectrometer, which operates in the IR region (3–12 μm) with 8 cm^−1^ spectral resolution and noise equivalent spectral radiance (NESR, in nW/cm^2^sr·cm^−1^ units) 7.5@3.8 μm, to enhance the capabilities of passive infrared surveillance. With the instrument, the data of several test panels, military vehicles and vegetated backgrounds at different times and under various environmental condition were obtained, their analysis of the experimental results statistically showed that the thermal sensor could detect the target hidden in vegetated and desert backgrounds with the use of multispectral techniques. As their work mainly focused on post-collection data analyses of infrared hyperspectral measurements and multispectral target detection algorithms, the design of the instrument, the real experimental image data and how the multispectral technology could be employed as an effective supplementary method for the conventional mid-infrared broadband thermal detection over the diurnal cycle was not mentioned. Nevertheless, their research results showed the potential and capacity of multispectral processing to detect low-contrast ground targets by providing valid estimates of targets to the background spectral contrast. 

Overall, from the abovementioned research results, although the polarization technique was an effective solution to thermal crossover detection, there were still some disadvantages. Firstly, the improvement of the infrared image contrast resulted from the calculation of Stokes vector formula, which means that the contrast enhancement is not direct. Secondly, the time division imaging or simultaneous imaging technique are usually used in polarization detection, which would increase image processing time or the system size and weight. In addition, the environmental factors could affect the polarimetric contrast. Potential sources include vehicles, buildings, trees, clouds, water vapor, etc., which are not necessarily visible within the scene but still illuminate the objects in the field of view of the detector could be a reduction in the magnitude of polarimetric signature of a target. Compared with the polarization technique, as the target’s infrared spectrum signature only differs with materials, one or some characteristic wavelengths could be enough to reflect the difference between the target and background without any redundant calculation. Thus, it would be faster and more direct to distinguish the target from the background in a complex environment with the multispectral technology if the characteristic wavelengths were acquired in advance according to prior knowledge. In this paper, our goal is to discuss how the multispectral technology could be employed to solve the problem of thermal crossover, design a fast, compact and light infrared multispectral prototype with the known characteristic wavelengths according to the prior knowledge and conclude that multispectral technology is capable of enhancing conventional thermal imaging.

### Overview of Thermal Detection over the Diurnal Cycle

**Figure 1:**
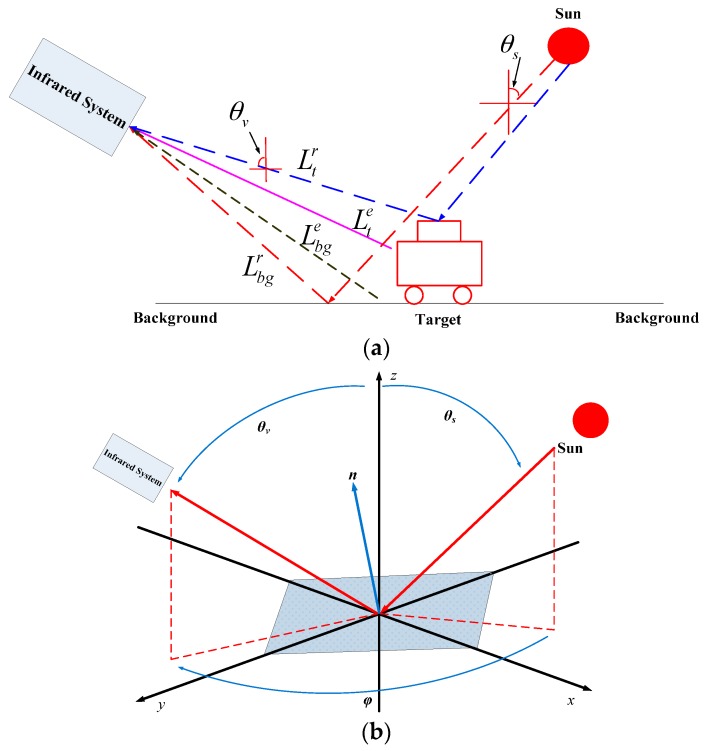
(**a**) Schematic of the infrared system measuring the radiance *L_t_* and the background radiance *L_bg_*. (**b**) Description of *θ_ν_*, *θ_s_* and φ.

Thermal crossover is defined as a natural phenomenon that normally occurs twice daily, but may occur at any time throughout the day when temperature conditions are such that there is a loss of contrast between two adjacent objects on the infrared sensor. Figure 1 pictorially shows a schematic of an infrared system measuring the target radiance *L_t_* and the background radiance *L_bg_*. The infrared system can be any conventional infrared sensor or camera and located at any arbitrary orientation. The target can be any typical common objects, such as vehicles, and the background can be any natural or artificial objects, such as grass, tree, or road. To simplify, without considering the scattering, the total received radiance at the infrared system can be expressed by two components:

(1) $$\left\{ \begin{array}{l} L_{bg}(\lambda ,\theta_{v},\theta_{s},\varphi )=L_{bg}^{r}(\lambda ,\theta_{v},\theta_{s},\varphi )+L_{bg}^{e}(\lambda ,\theta_{v},\theta_{s},\varphi ,T)\\ L_{t}(\lambda ,\theta_{v},\theta_{s},\varphi )=L_{t}^{r}(\lambda ,\theta_{v},\theta_{s},\varphi )+L_{t}^{e}(\lambda ,\theta_{v},\theta_{s},\varphi ,T) \end{array}\right.$$

where $L_{bg}^{e}$ and $L_{t}^{e}$ are the emissive radiance (the radiant flux emitted by a surface, per unit solid angle, per unit projected area, per wavelength) of the background and target, $L_{bg}^{r}$ and $L_{t}^{r}$ are the reflection of the solar irradiance on the background and the target, *λ* is the wavelength of light, *θ_ν_* is the viewing zenith angle of the detection system, *θ_s_* is the solar zenith angle, and φ is the azimuth angle between *θ_ν_* and *θ_s_*, T is the temperature. As DN=a⋅L+b, the *DN* difference between the targets and background objects (represented by C) can be expressed as [21]:

(2) $$C=\left|DN_{t}-DN_{bg}\right|=\left|a(L_{t}-L_{bg})\right|=a\left|\left(L_{t}^{e}-L_{bg}^{e})+(L_{t}^{r}-L_{bg}^{r}\right)\right|$$

Furthermore, assuming that the reflectivity of the target and the background objects are $\rho_{t}$ and $\rho_{bg}$, respectively, if ignoring the scattering and transmittance, the target and background objects’ absorptivity would be $\alpha_{t}=1-\rho_{t}$ and $\alpha_{bg}=1-\rho_{bg}$. As the vast majority of objects in nature produce diffuse reflection, the reflectivity $\rho_{t}$ and $\rho_{bg}$ should be replaced by the Bidirectional Reflectance Distribution Function (BRDF, a function which defines the spectral and spatial reflection characteristic of a surface and is the ratio of reflected radiance to incident irradiance at a particular wavelength [22]) to represent the anisotropic properties of solar radiation effects on the reflectivity of objects. Therefore, Equation (2) can be rewritten as:

(3) $$C=a\cdot \left|\begin{array}{l} \int_{\lambda_{1}}^{\lambda_{2}}\left[{BRDF}_{t}\left(\lambda ,\theta_{s},\theta_{v},\varphi \right)-{BRDF}_{bg}\left(\lambda ,\theta_{s},\theta_{v},\varphi \right)\right]L_{s}(\lambda )d\lambda \\ +\int_{\lambda_{1}}^{\lambda_{2}}\left(\left[1-{BRDF}_{t}\left(\lambda ,\theta_{s},\theta_{v},\varphi \right)\right]L_{t}^{e}\left(\lambda ,T\right)-\left[1-{BRDF}_{bg}\left(\lambda ,\theta_{s},\theta_{v},\varphi \right)\right]L_{bg}^{e}\left(\lambda ,T\right)\right)d\lambda \end{array}\right|$$

where $L_{s}(\lambda )$ is the solar radiation and $\lambda_{1}$~$\lambda_{2}$ is the working wavelength range of the infrared thermal sensor. In the case that *θ_ν_*, *θ_s_* and *λ* are constant, BRDF only differs with the object’s material.

**Figure 2:**
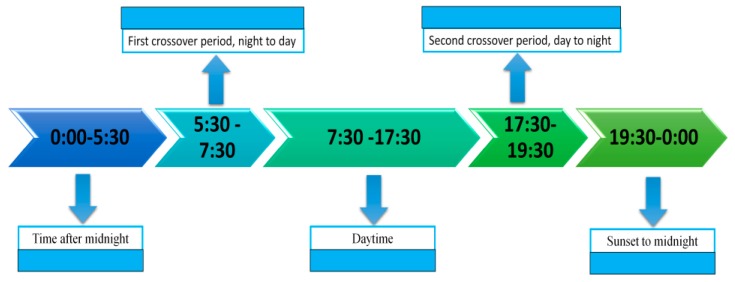
Five time zones from over one day.

As can be seen from Equation (3), in general, the thermal crossover over the diurnal cycle would occur when *C* between the target and the background is zero or below the threshold value required to execute a specific task by the conventional infrared thermal sensor. Specifically, we divide one day, 24 h, into five time zones, as shown in Figure 2.

Time after midnight:

(4) $$C=a\left|\int_{\lambda_{1}}^{\lambda_{2}}\left(\left[1-BRDF_{t}(\lambda ,\theta_{s},\theta_{v},\varphi )\right]L_{t}^{e}(\lambda ,T)-\left[1-BRDF_{bg}(\lambda ,\theta_{s},\theta_{v},\varphi )\right]L_{bg}^{e}(\lambda ,T)\right)d\lambda \right|$$

provided that the target and background have different material; in this case, the thermal crossover would not occur as *C* would not be zero.

First crossover period: in case of a sunny day, for the target with lower thermal inertia, such as metal, thermal crossover would occur. In this case, the multispectral exploration technique can be used to find the emissivity difference between the target and the background in Δλ to enhance the contrast *C*. For the target with higher thermal inertia, such as water, thermal crossover might not occur.

Daytime: The circumstance is more complicated as $L_{s}(\lambda )$ would have an effect on thermal crossover. No matter whether the target has lower or higher thermal inertia, thermal crossover may occur at any time, depending both on temperature differences and environmental factors, such as rain, and fog. In this case, the multispectral exploration technology can still be used to find the emissivity difference in Δλ to enhance the contrast *C* if thermal crossover occurs.

Second crossover period: similar to the “first crossover period”, for the target with lower thermal inertia the thermal crossover would occur and the multispectral exploration technology can be used to solve the problem of infrared detection during thermal crossover periods.

Sunset to midnight: similar to the “time after midnight”, provided that the target and background have different materials *C* would not be zero and thermal crossover would not occur.

## 2. Materials and Methods

### 2.1. Why the Infrared Multispectral Technology Works

From the abovementioned discussion and Equation (3), it can be seen that it is the combined impact of temperature difference, emissivity difference between the targets and background objects, and reflected solar radiation that leads to the occurrence of thermal crossover. To simplify the problem analysis, the single factor analysis of temperature and emissivity was specified in the following two cases.

In the first case, we assume that the targets and background objects have the same emissivity and use RRD(λ,T) to represent the relative thermal radiation differences between the targets and background objects, which is shown as Equation (5).

(5) $$RRD(\lambda ,T)=\frac{\frac{1}{\pi }\int_{\lambda_{1}}^{\lambda_{2}}\left(\alpha_{t}L_{t}^{e}(\lambda ,T_{1})-\alpha_{bg}L_{bg}^{e}(\lambda ,T_{2})\right)d\lambda }{\frac{1}{\pi }\int_{\lambda_{1}}^{\lambda_{2}}\alpha_{bg}L_{bg}^{e}(\lambda ,T_{2})d\lambda }$$

**Figure 3:**
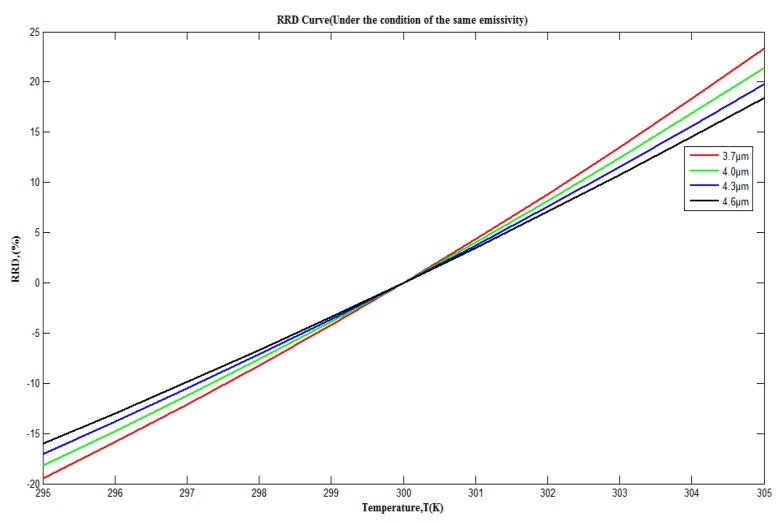
Relative thermal radiation differences Curve under the condition of the same emissivity in 3.7 μm–4.8 μm region.

Figure 3 shows the graphed outputs of Equation (5), provided that the ambient temperature was 300 K and the temperature difference between the targets and the background objects changes within ±5 K. As can be seen from Figure 3, between the 3.7 μm–4.8 μm region, which is also the typical working wavelength range for a commercial infrared detector, RRD(λ,T) changes within −20%–25% In addition, with the decrease of wavelength, the curve RRD(λ,T) becomes steeper and would be more sensitive to the changes in temperature. Particularly, the calculation of Equation (5) in the whole 3.7 μm–4.8 μm region was also made (not shown in Figure 3) and RRD(λ,T) changes within a smaller region, −15%–15%, which points out that, to a certain degree, for the traditional infrared broadband thermal sensor, compared to the one with several narrow wavebands, the thermal crossover would be more likely to happen and affect thermal detection for a longer time under the same conditions. 

**Figure 4:**
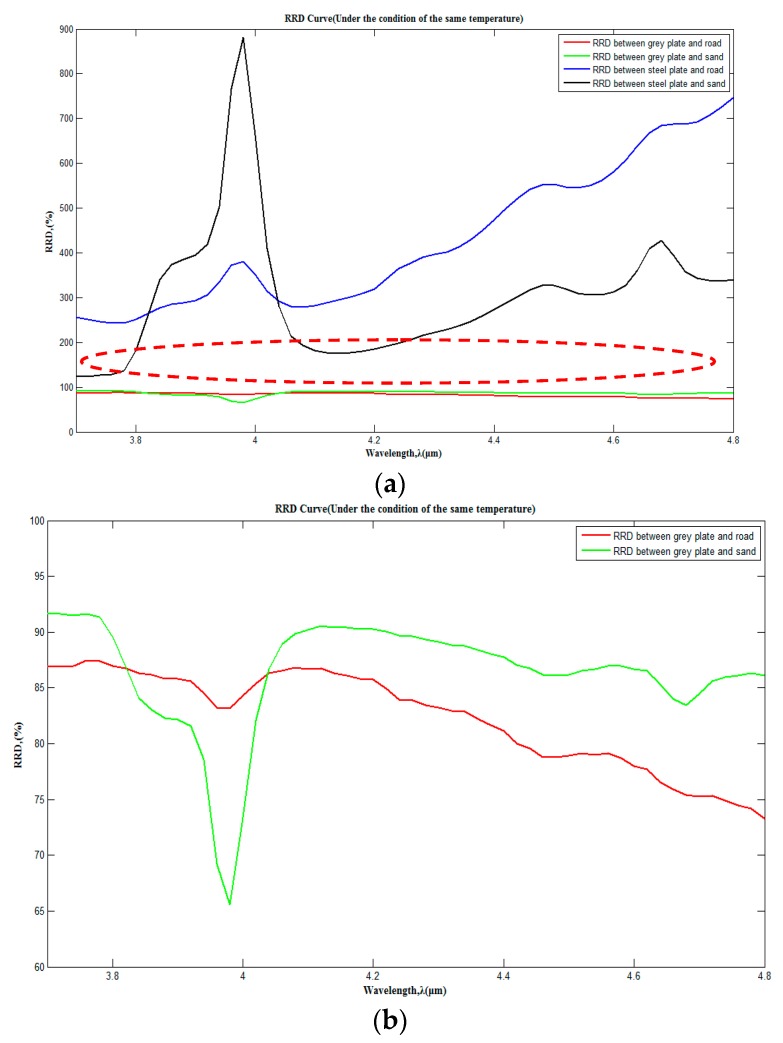
RRD curve under the condition of the same temperature in the 3.7 μm–4.8 μm region. (**a**) RRD curve among the targets grey plate, steel plate, and the background objects road and sand; (**b**) RRD curve among the target grey plate and the background objects road and sand.

In the second case, we assume that the targets are grey plate and steel plate, and background objects are road and sand, respectively, both of them have the same temperature, 300 K. With the emissivity data obtained from the IR module using the software Sensors, the calculation results of Equation (4) is shown as Figure 4. As can be seen from Figure 4, in the 3.7 μm–4.8 μm region, the RRD(λ,T) curve changes from 65% to 900%. Compared with RRD(λ,T) in the first case, obviously, the change of RRD(λ,T) caused by emissivity presents a greater volatility and wider range than that caused by temperature in Figure 3, which, in other words, indicates that the emissivity difference under characterized bands between the targets and background objects could be utilized to solve the detection problem during the thermal crossover periods.

### 2.2. Design of the Multispectral Infrared Imaging System

**Figure 5:**
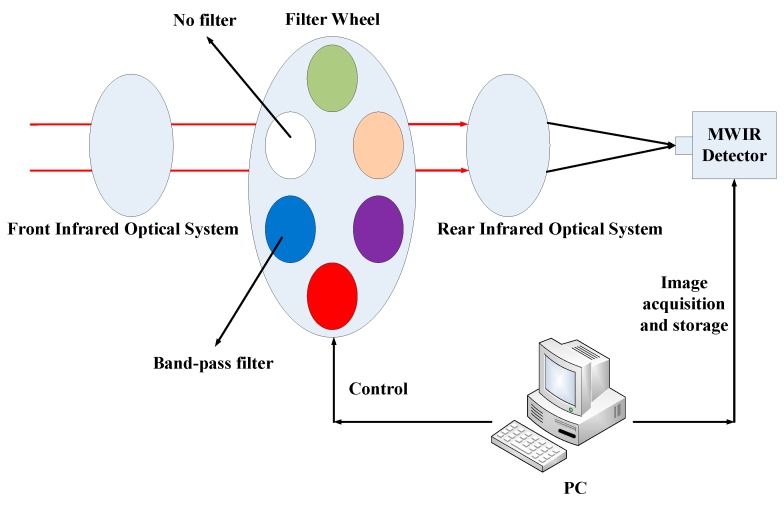
Schematic of multispectral infrared imaging system.

**Figure 6:**
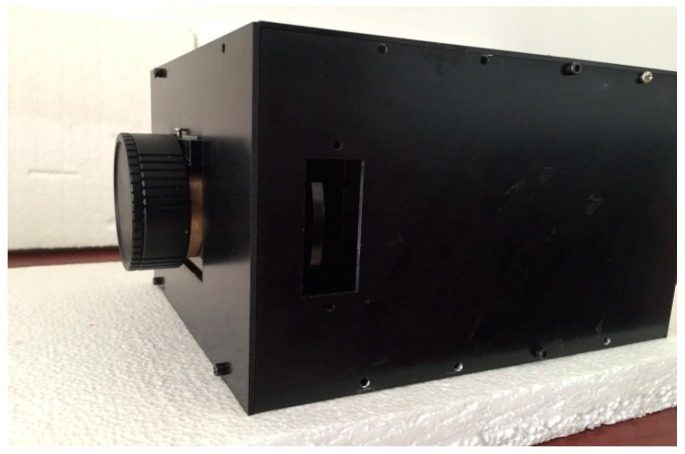
Prototype of multispectral infrared imaging system.

In order to verify the effectiveness of the multispectral infrared technology in solving the thermal crossover detection problem. A multispectral infrared imaging system prototype was designed and employed to conduct field experiment. The prototype consists of the infrared optical system, which is composed of the front infrared optical system and rear infrared optical system, a filter wheel with five band-pass filters, and a mid-infrared detector, as shown in Figure 5. The infrared camera lens has a focal length 100 mm. The mid-infrared detector is a France Sofradir Ltd. Model Mars 320 × 256 detector operating in region of 3.7–4.8 μm with a 5.5° × 4.4° field of view and up to 100 fps; this detector has a geometrical resolution of 0.3 mrad and a minimum detectable temperature difference between pixels of 0.03 °C and NETD of 9 mK. The five band-pass filters are produced by Sweden Spectrogon Ltd. with central wavelengths of 3700 nm, 3800 nm, 4120 nm, 4420 nm, and 4720 nm, respectively, and mounted on the filter wheel, which is driven by a stepper motor. In addition, the filter wheel reserves a hole without any filters so that the image comparison between the traditional broadband infrared image and narrowband infrared multispectral images can be conducted. Additionally, the cold reflection impact on the image has been considered and reduced to the minimum. The mid-infrared detector, filter wheel, and data acquisition and storage are controlled by a PC. The laboratory prototype is shown in Figure 6.

For the polarization technique used by Felton in [7,8,9], the MWIR imaging polarimeter employed division-of-aperture lens technology and the infrared image contrast improvement resulted from the calculation of Stokes vector formula. Thus, its image processing time, the system size and the system weight were longer and larger, the three parameters were 87 fps, the system size was 420L × 90W × 210H and weight was 4.99 kg. However, for our designed multispectral imaging system prototype, one or some characteristic wavelengths would be enough to reflect the difference between target and background without any redundant sensors and calculation if the characteristic wavelengths were acquired in advance according to the prior knowledge. Thus the prototype is faster, more compact, lighter and less costly. The image processing time of our prototype was up to 100 fps, the system size was 200L × 180W × 135H and was 3 kg, making it more suitable for practical applications. The specific specifications of the two sensors are listed in Table 1.

Prior to the field experiment, the infrared multispectral imaging system is radiometrically calibrated in the field laboratory through a calibration procedure developed by EOI Ltd. so that the imaging capabilities of each wavelength can be assessed. The imaging capabilities measurement results are summarized in Table 2.

When conducting reconnaissance or surveillance tasks with the infrared multispectral imaging system, the blank hole without any filters is initially rotated to the optical axis of the system and the imaging system is just a conventional broadband infrared sensor under the initial state. With the change of time and weather conditions around the observed area, thermal crossover may occur. Once the observed target is hidden in the background caused by thermal crossover, the PC will rotate the filter wheel and control the detector to acquire the images under different multispectral wavebands to find the emissivity difference in the narrow wavebands between the targets and background objects and solve the thermal crossover problem. Afterwards, in order to highlight the multispectral information of the image and conform to the human eye’s visual acquity at the same time, the narrow-band multispectral images and infrared broadband thermal image are blended to enhance the target recognition and solve the thermal crossover problem by using an HSV fusion algorithm [23]. HSV is one of the color systems that is used to pick a color from the color palette (H is hue, S is saturation and V is Value) and it is closer to people’s experience and perception of color, compared with RGB.

### 2.3. Consideration of Experiment Design

**Figure 7:**
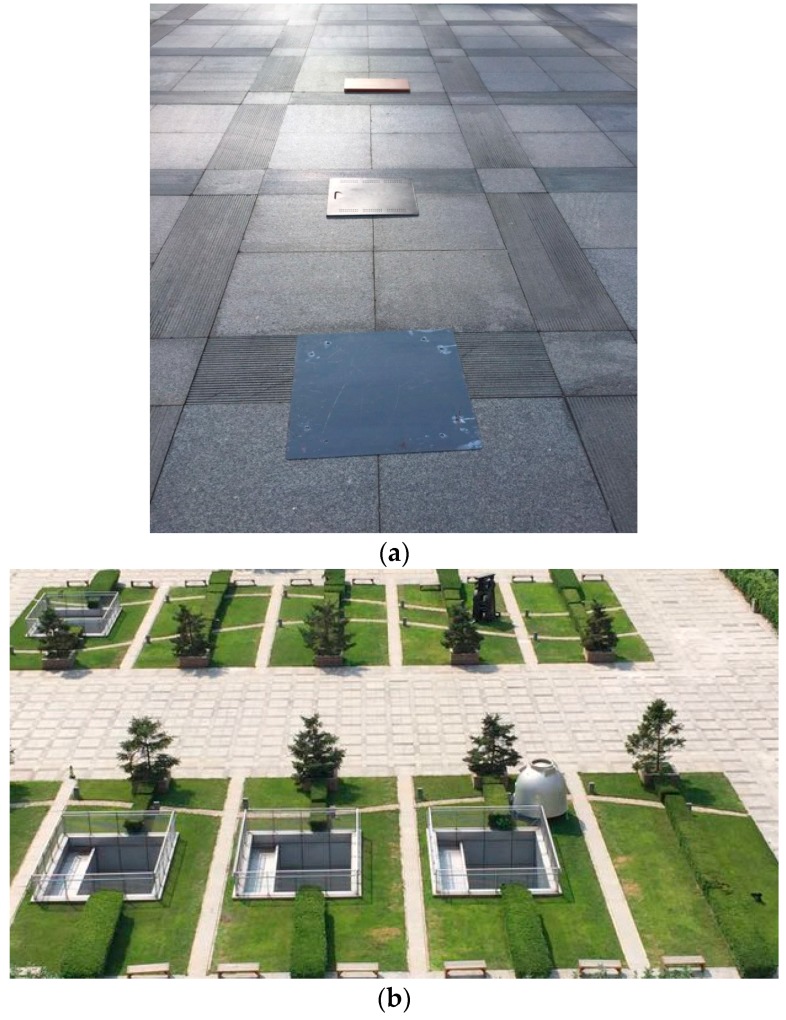
(**a**) Visible image of the target site consisting of three different plates and natural background; (**b**) The visible image of the test scene obtained on 15 March 2016.

To test the validity of the prototype, the field experiment was performed at the New Main Building at Beihang University. The infrared multispectral sensor was situated on the eighth floor of Tower B of the New Main Building (approximately 40 m) looking out of the window in the direction towards the target site, which was at approximately 100 m in distance. This open area was selected for the purpose of long-period image acquisition. The targets consisted of three different plates, galvanized sheet, steel sheet, and a wooden plate, and the natural backgrounds included grass, trees, and concrete road, which are shown in Figure 7a,b.

The wooden plate was selected to make comparative experiments in order to prove the thermal radiation difference between the object with high inertia and one with low inertia over the diurnal cycle. The galvanized sheet and steel sheet were selected to demonstrate the thermal radiation difference under their characteristic wavebands and confirm the effectiveness of the multispectral technology. As some environmental parameters, like ambient temperature, relative humidity, and solar irradiance, may affect the experiment result, the experiment date was selected in advance according to the weather forecast, and the environmental parameters with a large ambient temperature difference, fewer clouds during the testing period, and relatively stable humidity, are advantageous to the experiment. The environmental data was collected on 15 March 2016 and the image data and environmental parameters were acquired continuously between 00:00 on 15 March 2016 and 23:59 on 15 March 2016 with a speed of half a minute per image and half a minute per measurement, respectively. The sunrise and sunset on 15 March 2016 occurred at roughly 06:25 and 18:21, respectively.

## 3. Results and Discussion

**Figure 8:**
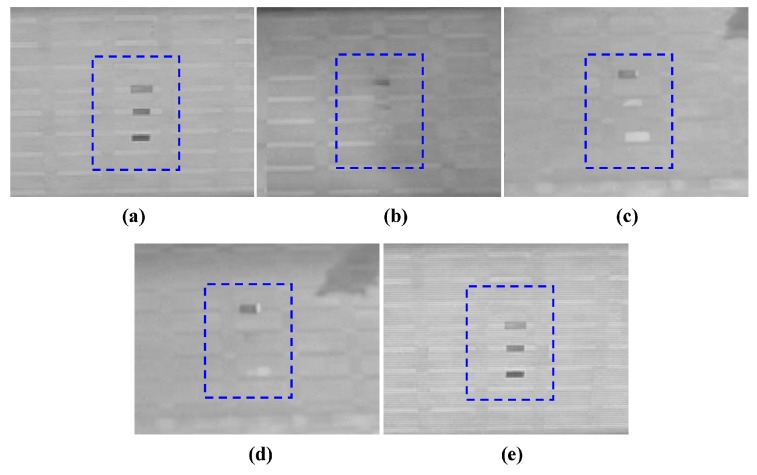
Infrared image obtained in each time zone. (**a**) Infrared image obtained at 03:00; (**b**) infrared image obtained at 06:50; (**c**) infrared image obtained at 12:30; (**d**) infrared image obtained at 18:05; and (**e**) infrared image obtained at 21:00.

According to Figure 2, in which the 24 h day was divided into five time zones, the experimental results were demonstrated similarly. The contrast ratio between the DN values of the target and the background $C^{\prime }=DN_{target}/DN_{bg}$ can be employed to reflect the contrast change of the regions of interest (ROI). At the time, after midnight, due to the different materials and temperature among the target galvanized sheet, steel sheet, and wooden plate, and the background road, $C^{\prime }$ did not approach 1 and thermal crossover did not occur, as shown in Figure 8a. At the first crossover period, for the wooden plate with higher thermal inertia, $C^{\prime }$ was 0.837 and thermal crossover did not occur, while for the galvanized sheet and steel sheet with lower thermal inertia, $C^{\prime }$ was approximately 1 (the exact number was 0.962 and 1.025, respectively) and thermal crossover did occur, as shown in Figure 8b. During the daytime, as $L_{s}(\lambda )$ had an effect on thermal crossover, the temperature difference among the background and the galvanized sheet and steel sheet increased gradually, $C^{\prime }$ was significantly greater than 1 and thermal crossover did not occur unless there was a rapid change in the weather conditions, as shown in Figure 8c. At the second crossover period, for the galvanized sheet and steel sheet with lower thermal inertia, $C^{\prime }$ was 1.011 and 1.045, respectively, and thermal crossover did occur again while, for the wooden plate, $C^{\prime }$ was 0.924 and the thermal crossover still did not occur, as shown in Figure 8d. From sunset to midnight, for each target $C^{\prime }$ was far less than 1 and thermal crossover did not occur, as shown in Figure 8e. Additionally, the average grey value of the image was larger than Figure 8a at the time after midnight because of the higher temperature. The contrast values among the three targets and the background in Figure 8 are listed in Table 3.

**Figure 9:**
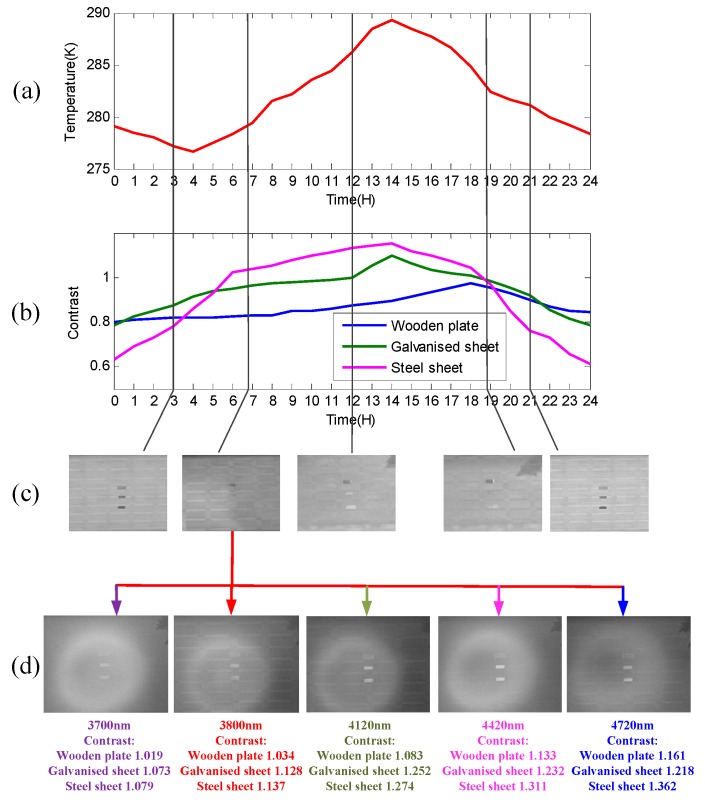
(**a**) Ambient temperature during the 24-h test; (**b**) contrast curve among the three targets and the background road; (**c**) images of the targets in the five time zones; and (**d**) images of the targets at 3700 nm, 3800 nm, 4120 nm, 4420 nm, and 4720 nm at the first diurnal cycle.

In order to clarify the effectiveness of the multispectral technology to solve the thermal crossover problem, the multispectral images under central wavelengths of 4120 nm, 4420 nm, and 4720 nm at the first crossover period were obtained, and the results are presented in the form of image contrast plots, calculated using $C^{\prime }=DN_{target}/DN_{bg}$. Included with each of these plots are the corresponding environmental data, as shown in Figure 9. Specifically, Figure 9b,c clearly show that the contrast curve of the galvanized sheet and steel sheet varied more significantly than the wooden plate during the 24-h test period and the contrast of the galvanized sheet and steel sheet was close to 1 during two diurnal cycles, while the contrast of the wooden plate fluctuated between 1.0 and 1.16 throughout the experiment time, proving the existence of thermal crossover for the objects with lower thermal inertia once again. Figure 9d showed the multispectral images, which were obtained at the same period with Figure 9b. As can be seen from Figure 9d, the multispectral technology was used to find the emissivity difference between the target and the background at 3700 nm, 3800 nm, 4120 nm, 4420 nm, and 4720 nm to enhance the contrast among the galvanized sheet, steel sheet, and road. Among the five wavebands the best contrast improvement was at 4720 nm, with 4420 nm following, which presented a consistent trend in accordance with Figure 4a and indicated the effectiveness of the multispectral technology in solving the thermal crossover problem. The contrast values among the three targets and the background under different wavebands in Figure 9d are listed in Table 4. Compared with the Figure 8, the image contrast enhancement in the target area is direct after employing the narrow band-pass filters.

**Figure 10:**
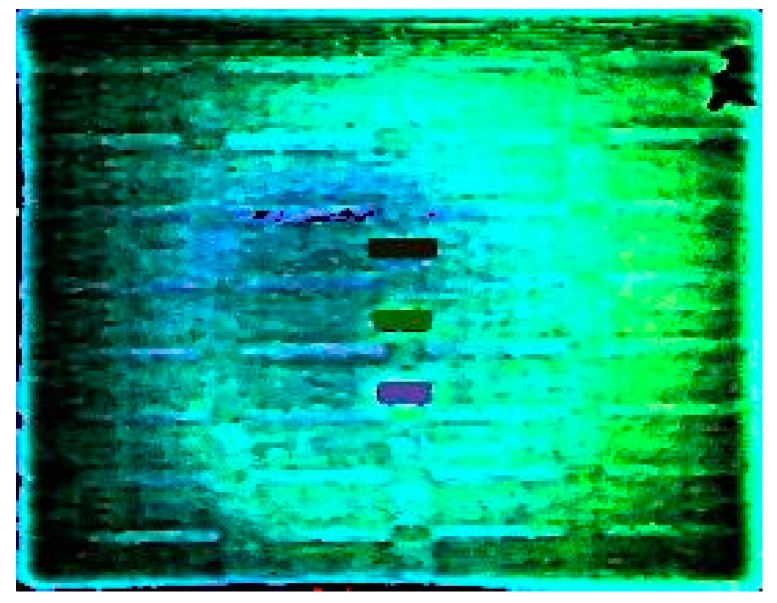
Pseudo-color image fused by Figure 7b and Figure 8d.

Figure 10 showed the pseudo-color image obtained by running the HSV image fusion algorithm described in Section 3 with Figure 8b and Figure 9d. Combining the infrared broadband image with the infrared images under characteristic wavebands, the three targets were marked with different colors and presented clearly, by which the multispectral technology employed an effective supplementary method for the conventional mid-infrared broadband thermal sensor to solve the thermal crossover detection problem.

Further, it can be noted that the magnitude of contrast improvement is not as large as the calculation results in Figure 4a because of the solar radiation effect, stray radiation caused by band-pass filters, and the difference between the actual emissivity value and the real emissivity value. However, this does not influence our experimental conclusions that multispectral technology can be employed to solve the thermal crossover problem.

**Figure 11:**
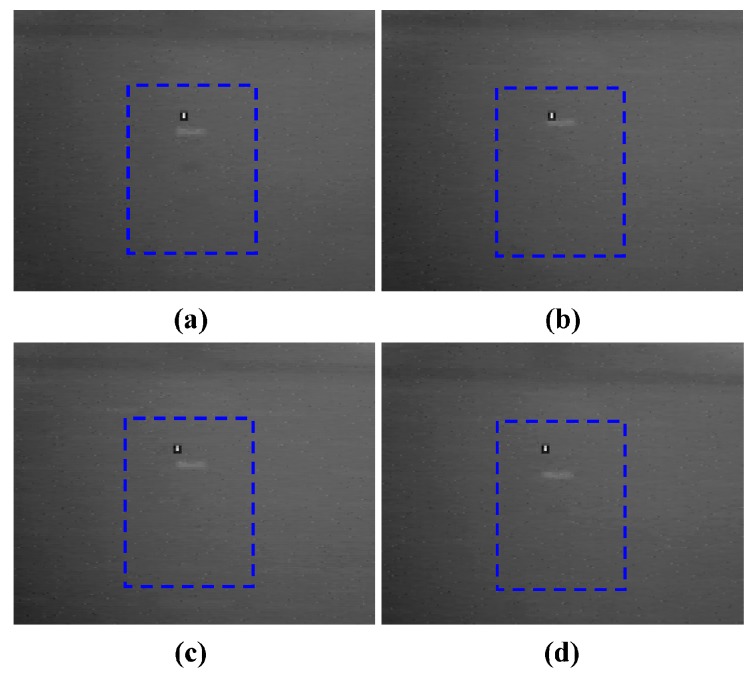
Infrared polarization images with four polarization states. (**a**) 0°; (**b**) 45°; (**c**) 90°; (**d**) 135°.

In order to further show the advantage of multispectral technology in solving the thermal crossover problem, the same field experiment with polarization technique by using 0°, 45°, 90°, 135° four linear polarizers was also conducted and the four polarization state polarization images obtained at 07:00 were shown in Figure 11.

As can be seen from Figure 11, for the wooden plate, thermal crossover still did not occur, while for the galvanized sheet and steel sheet, the thermal crossover did occur, which was similar to the results with multispectral technology. The contrast values between the galvanized sheet, steel sheet and the background in Figure 11 are listed in Table 5.

**Figure 12:**
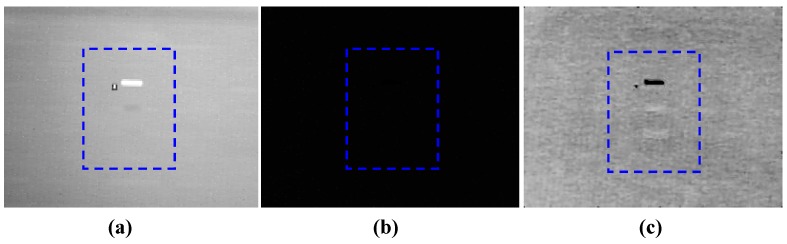
S_0_ and S_1_ Stokes parameter images (**a**) S_0_ image; (**b**) S_1_ image; (**c**) S_1_ after contrast stretching.

From Table 5, it can be found that, compared with the infrared multispectral images, without further image processing, the image contrast enhancement in the target area in the infrared polarization images with four polarization states were not obvious. Thus, the Stokes vectors, which completely characterized the polarization states of targets from the scene need to be calculated. The data products used in this experiment included S_0_ and S_1_ Stokes parameter images where S_0_ is the horizontal (0°) plus the vertical (90°) components of polarization and the S_1_ Stokes parameter is the horizontal minus the vertical components of polarization. The S_0_ and S_1_ Stokes parameter images are shown as Figure 12a,b respectively.

The image contrast of the galvanized sheet and steel sheet in Figure 12a was 0.921 and 1.073, respectively, which showed some extent of improvement compared with Figure 11. However the DN difference between the galvanized sheet, steel sheet targets and background in Figure 12a were only 16 and 7. In Figure 12b, although the calculated image contrast, according to $C^{\prime }=DN_{target}/DN_{bg}$, was improved, it was meaningless as the DN of targets and background were too low to be sensed by eyes. In fact, the DN difference between the galvanized sheet, steel sheet targets and background in Figure 12b were only 3 and 2, respectively. In order to show the targets in Figure 12b relatively clearly, the images were further processed with the contrast stretching algorithm, as shown in Figure 12c. Through the data processing procedure, it could be found that even with the Stokes parameter calculation, the difference between target and background had still not been improved significantly so that further image processing procedures were required. The main reason for the polarization detection experiment result was that the abundant geometry information contained in the background weakened the polarization characteristics differences. Because the polarization technology achieves distinction between target and background through the perception of their polarization characteristics differences, the background information might have an influence on the target detection. However, for the multispectral technology, as stated previously, compared with the polarization technique, as the target’s infrared spectrum signature only differs with materials, it would be faster and more direct to distinguish the target from the background in the complex environment only if the characteristic wavelengths of the targets and backgrounds were acquired in advance.

## 4. Conclusions

As the thermal crossover has great influence on the infrared sensors working in a single wide range, it is significant to solve this problem for the conventional mid-infrared thermal sensor, especially for the infrared surveillance system. In this study, we analyze theoretically how the thermal crossover disables the conventional thermal sensor and under what conditions the thermal crossover would happen. Furthermore, based on the analysis, a fast, compact and light optical prototype based on infrared multispectral technology is designed with the known characteristic wavelengths according to the prior knowledge. Then the experimental process has been optimized and more image data is provided, especially regarding what the pictures are when the target integrated with the background during the diurnal cycle. Then, the whole process of employing the multispectral technology to solve the thermal crossover detection problem is clearly shown. In addition, a comparison experiment with polarization technique is also conducted to further show the advantage of multispectral technology. 

The field experiment with multispectral technology was conducted over a 24-h period with the targets of galvanized sheet, steel sheet, and wooden plate, and the background road on a sunny day. The results showed that, for the galvanized sheet and steel sheet targets, the thermal crossover could affect a contrast for up to four hours at two diurnal cycles, jeopardizing the success of surveillance missions. For the wooden plate target, although the image contrast reduced over the diurnal cycle, it could still distinguish the targets from the background objects, which means that thermal crossover might not always occur, or even possibly not exist at all over the diurnal cycle for the objects with higher thermal inertia. Through employing the infrared narrow band-pass filters, thermal crossover in the first diurnal cycle was relieved as the contrast was upgraded to the levels such that the metal targets could be distinguished from the background objects. Furthermore, the experimental results provided us with the information about what the characterized bands between the targets and background objects were, which would be useful for system design in the future. In addition, the pseudo-colored image produced by multi-spectral image fusion method showed the effectiveness of the multispectral technology for contrast promotion of each target. Then, as a comparison, the same field experiment with polarization technique by using 0°, 45°, 90°, 135° four linear polarizers was also conducted and the S_0_ and S_1_ Stokes parameter images showed that the image contrast showed some extent of improvement but no obvious improvement, as the background weakened the polarization characteristics differences.

While promising, the field experiment should just be considered as very preliminary practical application and the experimental results should also just be viewed as a proof-of-principle. Nevertheless, the conclusion that the multispectral technology can be employed to solve the thermal crossover problem is unambiguous. In future, it might be possible to further extend the range of applications for the conventional thermal infrared broadband sensor into the thermal crossover periods by exploiting the emissivity of infrared spectral signatures and fusing multispectral images from the perspective of mid-infrared thermal detecting system design. Research focusing on the characterized bands between different common targets and background objects and how the weather conditions influence the thermal crossover will be undertaken.

## Figures and Tables

**Table 1:** Specifications of imaging polarimeter and multispectral infrared thermal sensor.

Parameter	Value of Felton’s MWIR Imaging Polarimeter	Value of Our MWIR Imaging Spectrometer
Technological type	Polarization technique with four polarizers	Multispectral technique with five filters
FOV (°)	5.5	5.5 × 4.4
Focal Length (mm)	100	100
F/#	2.3	2
Total FPA pixels	Four FPA arrays 640 × 512 (single 220 × 220)	Single FPA 320 × 256
Pixel size (μm)	24 × 24	30 × 30
Max Frame Rate (fps)	87	up to 100
Sensor Dimensions (mm)	420L × 90W × 210H	200L × 180W × 135H
Sensor weight (kg)	4.99	3
Central wavelength (nm)	-	3700 nm, 3800 nm, 4120 nm, 4420 nm, 4720 nm
FWHM (nm)	-	80~100 nm
Sensitivity	10^−7^ W/cm^2^sr	9 mK

**Table 2:** Noise equivalent temperature difference (NETD) Measurement Results.

Wavelength	NETD (Background 298 K, f/2, Integration Time 6 ms)
3700 nm	476.6 mK
3800 nm	556.2 mK
4120 nm	533.9 mK
4420 nm	513.6 mK
4720 nm	490.1 mK

**Table 3:** Contrast among the three targets and background in Figure 8.

Figure Number	Wooden Plate	Galvanized Sheet	Steel Sheet
a	0.822	0.784	0.632
b	0.837	0.962	1.025
c	0.825	1.098	1.151
d	0.810	1.011	1.045
e	0.807	0.786	0.612

**Table 4:** Contrast among the three targets and the background in Figure 9.

Wavebands	Wooden Plate	Galvanized Sheet	Steel Sheet
3700 nm	1.019	1.073	1.079
3800 nm	1.034	1.128	1.137
4120 nm	1.083	1.252	1.274
4420 nm	1.133	1.232	1.311
4720 nm	1.161	1.218	1.362

**Table 5:** Contrast between galvanized sheet, steel sheet targets and background in Figure 11.

Figure Number	Galvanized Sheet	Steel Sheet
a	0.909	0.942
b	0.977	0.964
c	0.979	0.981
d	0.965	0.976

## Abbreviations

The following abbreviations are used in this manuscript:

mmW	Millimeter Wave
NESR	Noise Equivalent Spectral Radiance
NETD	Noise Equivalent Temperature Difference
DN	Digital Number
BRDF	Bidirectional Reflectance Distribution Function
RRD	Relative thermal radiation differences
IR	Infrared
PC	Personal Computer
